# Consciousness as a Product of Evolution: Contents, Selector Circuits, and Trajectories in Experience Space

**DOI:** 10.3389/fnsys.2021.697129

**Published:** 2021-10-20

**Authors:** Thurston Lacalli

**Affiliations:** Biology Department, University of Victoria, Victoria, BC, Canada

**Keywords:** qualia versus formats as contents, neural correlates of consciousness, neural algorithms, topological representations, configuration spaces

## Abstract

Conscious experience can be treated as a complex unified whole, but to do so is problematic from an evolutionary perspective if, like other products of evolution, consciousness had simple beginnings, and achieved complexity only secondarily over an extended period of time as new categories of subjective experience were added and refined. The premise here is twofold, first that these simple beginnings can be investigated regardless of whether the ultimate source of subjective experience is known or understood, and second, that of the contents known to us, the most accessible for investigation will be those that are, or appear, most fundamental, in the sense that they resist further deconstruction or analysis. This would include qualia as they are usually defined, but excludes more complex experiences (here, formats) that are structured, or depend on algorithmic processes and/or memory. Vision and language for example, would by this definition be formats. More formally, qualia, but not formats, can be represented as points, lines, or curves on a topological experience space, and as domains in a configuration space representing a subset of neural correlates of consciousness, the selector circuits (SCs), responsible for ensuring that a particular experience is evoked rather than some other. It is a matter of conjecture how points in SC-space map to experience space, but both will exhibit divergence, insuring that a minimal distance separates points in experience space representing different qualia and the SCs that evoke them. An analysis of how SCs evolve over time is used to highlight the importance of understanding patterns of descent among putative qualia, i.e., their homology across species, and whether this implies descent from an ancestral experience, or ur-quale, that combines modes of experience that later came to be experienced separately. The analysis also provides insight into the function of consciousness as viewed from an evolutionary perspective, defined here in terms of the access it allows to regions of SC-space that would otherwise be unavailable to real brains, to produce consciously controlled behaviors that could otherwise not occur.

## Introduction

Investigating the nature of consciousness is tricky exercise, a good part of which revolves around the hard problems and explanatory gaps beloved of philosophers (Levine, 1983, 2009; Chalmers, 1995; Van Gulick, 2018). This account is less concerned with those issues, i.e., consciousness as a phenomenon, than with the nature of consciousness as a product of evolution. More specifically, the issue here is a practical one, of finding a conceptual framework for dealing with the action of natural selection on the neural circuits that underpin conscious experience (here, by convention, simply “experience”), and how changes to the circuitry change the experience. How neural circuits evolve is a complex issue in its own right (Tosches, 2017). Adding consciousness to the mix is even more problematic, and perhaps uniquely so, in that we have no way as yet to identify the neural circuits responsible for evoking conscious sensations, and no way beyond inference to assess consciousness in taxa other than our own. But there is no justification for supposing *a priori* that a systematic reductionist approach will not eventually succeed in unraveling the mysteries of consciousness as it has with so many other natural phenomena.

Complex systems of interacting components clearly can have unexpected properties with the potential to provide a source for evolutionary innovation (Solé and Valverde, 2020), and this feature has been used to advantage in a number of theories of consciousness, including integrated information theory and some variants of computational, global workspace and higher order theories (Dehaene and Naccache, 2001; Piccinini and Bahar, 2013; Oizumi et al., 2014; Brown et al., 2019). And indeed, if vertebrate consciousness is entirely a product of cortico-thalamic circuitry, a widely accepted view (Butler, 2008), then complexity would seem to be inextricably linked with consciousness of any kind. Here, in contrast, my assumption is that, like everything else in evolution, complex forms of consciousness are more likely than not to have evolved from simple antecedents that were progressively elaborated and refined over an extended period of evolutionary time in ways that can be understood step by step in adaptive terms. This supposition is receiving increasing attention (Baron and Klein, 2016; Feinberg and Mallatt, 2016; Godfrey-Smith, 2016; Lacalli, 2018), and there is a recognition that even quite early vertebrates may play a role in the story if brain structures evolutionarily older than neocortex are involved, as has been argued for olfactory centers (Shepherd, 2007; Merrick et al., 2014; deVries and Ward, 2016), the optic tectum (Feinberg and Mallatt, 2016), subcortical telencephalic centers, and nuclei in the thalamus and midbrain (Merker, 2005, 2007, 2013; Ward, 2011; Woodruff, 2017). We would then have a much-expanded evolutionary window, materially increasing the prospects of finding vestiges of early stages in the transition from consciousness as it first emerged in evolution to something more complex. The cortex in such scenarios then appears in a different light, as less a precondition for having consciousness of any kind, than a device for exploiting more fully a capability the brain may already have possessed.

What approach should one then take when investigating consciousness from an evolutionary perspective? Consider the skeleton, another complex product of evolution: it consists of diverse parts, each precisely shaped to a purpose and assembled in a way that allows that assemblage to function effectively as a whole. By analogy, the diverse parts from which evolved consciousness is constructed are its distinguishable contents, and the evolutionary questions one can ask about these concern the role each part plays in the whole, and the means by which the whole is coordinated. This presupposes also that the contents of consciousness can be dealt with individually, as entities, and investigated as such. For my purposes I assume this to be the case. Accepting the counterargument, that consciousness is indivisible (e.g., Dainton, 2000; Tye, 2003), leads to a very different analytical focus. From an evolutionary perspective, the unity of consciousness is far more likely to be adaptive rather than intrinsic, in other words a secondary feature, refined progressively and of necessity because no product of evolution is of any use unless its constituent parts operate together in a coordinated way.

The analysis developed here focuses on selected individual contents, and is directed at the question of evolutionary change in general terms, rather than the pros and cons of any particular evolutionary scenario. Issues concerning the hard problems as usually defined are deferred because, from an evolutionary perspective, it is not important what consciousness “is” or from what it originates, only that it is useful (Lacalli, 2020, see Kostic, 2017 for a philosophical justification). As to why consciousness is useful, there will be both specific answers that highlight the relative advantages of conscious decision-making over reflex action in a given behavioral context (Velmans, 2012; Black, 2021), and a general answer that relates to the access consciousness provides, through the evolutionary process, to circuitry variants and behavioral outcomes that could otherwise not exist, as discussed in the concluding section (section “Conclusions, and the Function of Consciousness”).

**FIGURE 1:**
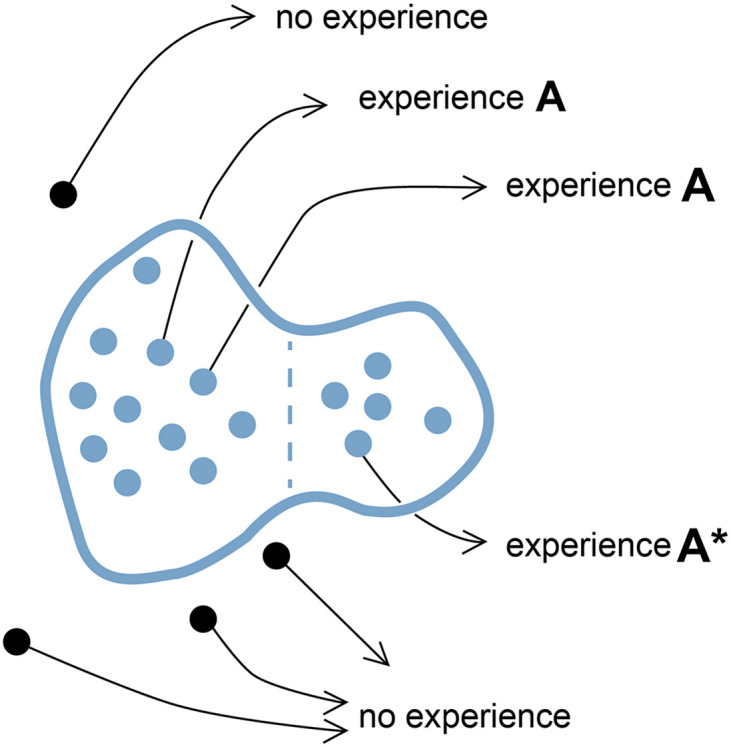
A point cloud in SC-space. The diagram represents a configuration space where distance measures incremental differences in the way a related set of neural circuits (variants) can be configured or, for the case of neural events, incremental differences in variables chosen to represent those events. Each point within the domain bounded in blue represents a selector circuit (SC), defined as a neural circuit or neural event that acts to evoke a particular experiential quale (the **A**s in this case). Black points outside the domain represent circuit or event variants that fail to evoke that quale, or in this case, any experience at all. For an individual brain, the quale in question could theoretically be evoked by many SCs acting in concert, represented here by a point cloud, or few, or only one. Individual brains could thus vary both quantitatively (how many variants are active and effective) and qualitatively (how tightly clustered they are in SC-space). Experience A* is included to indicate that there may be differences in the experience evoked at different points within the domain, i.e., that experience A* may be qualitatively different than **A**. For example, the domain as a whole might include SCs for both fear (**A**) and panic (A*), so a point cloud clustered predominantly on the left would evoke fear, on the right, panic, or both feelings together if the SCs are evenly distributed. How gradual the transition might be from fear to panic along trajectories in SC-space is not specified, nor how abruptly either experience is degraded for SCs located near the domain boundary, which could for that reason be “fuzzier” than shown here. The domain could also be more cloud-like in being diffuse and full of holes representing SCs inside the domain that happen not to produce an experience. Indeed, the term point cloud would typically be employed topologically to refer to the domain itself, so as to include the total set of all possible SCs of a specified type, but is here used in a more restricted sense, to refer to only those SCs realized in real brains at either the individual or population level. The diagram is highly schematic in reducing the high-dimensional space required to represent the complexities of real neural circuits and events to a two-dimensional surface, and is intended to apply only to the most fundamental units of experience, i.e., qualia.

**FIGURE 2:**
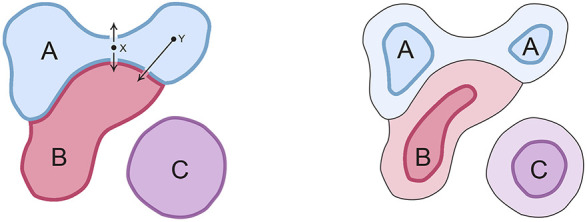
How evolution acts to select the subset of SCs realized in real brains. Consider hypothetical domains A and B within whose boundaries the respective SCs evoke two distinguishable experiences, **A** and **B**. For SCs in real brains, and assuming SCs change location from generation to generation due to genetic and developmental variability, short trajectories that change or abolish an experience (e.g., X in the figure) are more likely to occur than longer ones (Y). Subjective experiences are therefore less robust in evolutionary terms when the SCs that evoke them are close to domain boundaries. Hence, over evolutionary time, the region within which point clouds are realized (bounded domains on the left hand diagram) will progressively shrink and separate from one another (right hand diagram) as the SCs in intervening regions of SC-space (paler colors) are eliminated from real brains. Domain C is included as a reminder that multiple domains can act together, as ensembles, so that, for example, experience **A** might only be evoked if both A and C (plus any number of additional domains) act in concert, or A and C might together evoke an entirely different experience.

**FIGURE 3:**
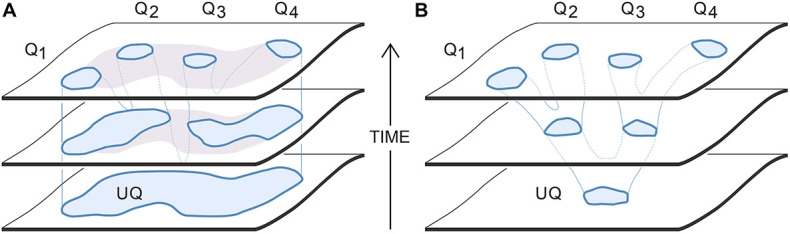
Two options for how a set of domains on SC-space, representing the SCs that evoke specific qualia (the Qs), could derive by common descent from those evoking a single ur-quale (UQ). The intent is to show how the ur-quale is changed in character (horizontal axis) over evolutionary time (vertical axis). **(A)** The evaporating puddle scenario: this assumes an ur-quale whose SCs occupy a large domain, which is not then precisely defined at first with regard to the experiences those SCs evoke. A range of sensations would hence be evoked together from which the descendant qualia are progressively refined. Since the SCs remain within the parent domain, each newly evolved quale would incorporate elements present in the ur-quale. **(B)** The branched tree scenario: this assumes the SCs evoking the single ur-quale were distinct and well defined from the start, so the initial point cloud would have been restricted to a smaller domain compared with the puddle scenario. Since the branches of the tree diverge, all of the Qs in the tree scenario (in this example, all but Q3) will differ qualitatively from the ur-quale from which they all derive. See text for details.

**FIGURE 4:**
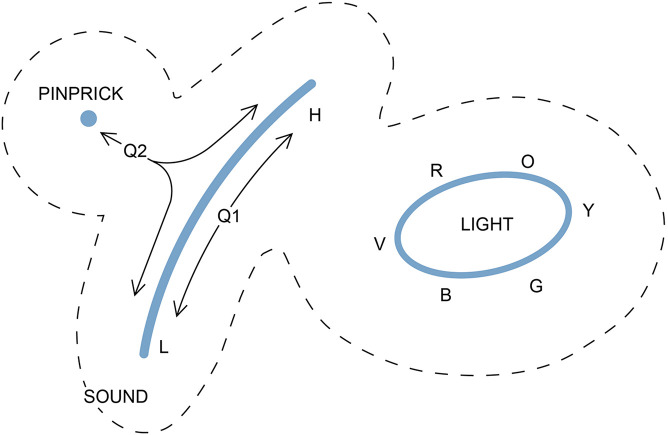
A way of representing three experiences on a two-dimensional E-space. A pinprick is suggested here as a simple example of tactile experience, disregarding its localization, that can be represented as a point. The sensation of itch might be equally suitable. Sound, for animals that can distinguish frequencies, would be a line from low (L) to high (H) frequency. Color, as we experience it, is a closed curve, as the sequence from red to orange, yellow, green, blue, and violet (R, O, Y, G, B, V) is recursive, leaving the center of the curve for their blended combination, white light. The dotted lines are a reminder that, if these experiences are to be plotted together, there must be a zone of exclusion between them that is devoid of realized experience, as the three experiences would otherwise risk being combined in ways that would render them less distinguishable. The diagram could well have looked quite different if we consider the evolutionary past, how the three qualia originated, and the degree of homology between them. This is shown by the trajectories (arrows from Q1 and Q2). Trajectories originating at Q1 show a route by which a frequency-dependent acoustic experience might have evolved from an ur-quale that originally produced a much more limited range of that experience. Trajectories radiating from Q2 show routes by which an ancestral ur-quale common to multiple mechanosensor-based experiences might have evolved so as to separately evoke sound and tactile sensations, making these homologous as mechanosensations. The trajectories represent sequences of states that have changed over time, but points along the Q2 trajectories are ones that would have been present only in past brains, not present ones, as the SCs responsible for evoking intermediates between the qualia in question would long since have been extinguished by selection. For qualia unrelated through homology as experiences, there may be no such intervening points, and hence no access to intermediate experiences. This could be the case for light and sound for example, which share no obvious qualitative features, in which case there would be no justification for even trying to map them to the same surface. The reader is encouraged to think about how the figure might be used to illustrate the differences between a puddle-like evolutionary sequence and a tree-like one, i.e., to construct an E-space counterpart of Figure 3.

A second set of questions concerns what can be said about the way the neural correlates of consciousness (NCCs) and the sensations they evoke will themselves evolve. These are explored below in a set of thought experiments, using two hypothetical spaces, one for neural circuitry (SC-space, described in the section “Selector Circuits: Robustness and Routes to Innovation”) and the other for subjective experience (E-space, described in the section “Trajectories in Experience Space”). The exercise is topological in a general way, with SC-space conceived of as a configuration space (Figures 1–3) and E-space as its non-physical counterpart (Figure 4). This choice limits the analysis to the simplest of contents (as explained in the section “Categorizing Contents”) in order to avoid the methodological problems of dealing with sequential processes, which for a topological approach might employ graph theory or recurrent neural networks, the latter being currently a favored model of choice (Schmidhuber, 2015; Yu et al., 2019). The exercise as a whole has practical value given the prospect that, through a combination of innovative optogenetic, 3D reconstruction and electrical recording tools (e.g., Marques et al., 2019; Abbott, 2020), an increasing amount of data relating to NCC activity can be expected in the not-to-distant future. In consequence, it is timely to begin thinking about what such data may reveal, and how they are to be analyzed. Topological methods are used elsewhere in the study of consciousness (e.g., Clark, 1996, 2000; Matthen, 2005; Rosenthal, 2010; Raffman, 2015), but not for the purpose of modeling evolutionary change.

## Categorizing Contents

It is important first to distinguish contents of consciousness that are suitable for the analysis that follows from those that are not. To avoid any confusion, the term “contents” is not meant here to refer to anything more mysterious than a list written down on a piece of paper, and in no way implies that consciousness has the properties of a vessel that needs filling, or is limited in what it can contain. Though these both may be true, they are irrelevant to the analysis. The relevant point is that the contents of consciousness vary in complexity, from simple sensations, like the sharp pain from the prick of a needle or the feeling of pleasure, anxiety or fear, to the visual, auditory and cognitive experiences of such activities as hunting prey, avoiding predators, or comprehending a lecture on cognitive neuroscience. Since my concern here is with the elaboration of experience from simple beginnings, the analysis is restricted to those contents that might reasonably be supposed to have emerged early in evolution, and hence were available to be employed as components of later evolving, more complex contents. To this end I make following conjecture: that much as molecules are constructed of atoms, complex experiential contents are constructed of multiple elements among which are more fundamental units that are themselves contents, but are irreducible. So, to continue the analogy, molecules are reducible by chemical means while atoms are not, hence the most fundamental units of consciousness, whatever those are, will be those that involve no procedural sub-processes, and resist deconstruction by any means we currently have at hand, whether verbal argument, physical intervention or mathematical analysis. In consequence, they cannot be apprehended except by direct experience, which makes them essentially equivalent to qualia as usually defined (Tye, 1995, 2018). I use the term here despite its detractors (see Kanai and Tsuchiya, 2012 for a defense) because a quale simply “is” and so is ineffable, like the classic example of perceiving the color red, which exactly suits my requirements.

The idea that qualia are fundamental units of experience is widespread in consciousness studies,^1^ but I treat them here as fundamental also for purposes of analysis and as objects of selection. Investigating consciousness from an evolutionary perspective has its own focus and agenda (Lacalli, 2021), and neither have been well served by existing theory. Addressing the question of what form consciousness took early in its evolutionary history is difficult to say the least, but is essential if we are ever to understand the link between consciousness as we experience it and the ancestral condition from which that consciousness derives. The current paper represents an attempt to do precisely that, but the methodology adopted is only directly applicable to a subset of experiences, namely those provisionally identifiable as qualia. For many theories of consciousness the focus is as much if not more so on complex contents, i.e., those combining qualia with other products of neural activity. Vision exemplifies this greater level of complexity, as the visual display, which allows the whole of the visual field to be perceived at once, has an intrinsic geometry and viewpoint that can be analyzed and understood in its own terms (Merker, 2007, 2013; Williford et al., 2018). One can then reasonably suppose that the properties of the display arise at least in part from the way visual input is processed and integrated, which will involve procedural rules, and so is sequential, algorithmic, and by analogy, computational (Wood, 2019). Hence the perception of a visual field, as an experience, is not a fundamental unit of consciousness as defined above, and its dependence on neural circuits and patterns of activity make it too complex to be represented by a configuration space. Contents of this type, which are beyond the scope of this analysis, will be referred to as “formats.” This would include vision, which, as a total experience, is a format. Similarly, memory dependence (Wilson and Sullivan, 2011) makes olfaction a format, though the NCCs responsible for evoking individual odors could potentially be mapped to a configuration space. Language would also be a format, for both its intrinsic structure and memory dependence (Chomsky, 1990; Jackendoff, 2002; Pinker and Jackendoff, 2005), as would everything that flows from the use of language, including reasoning, logic, and any form of conscious awareness with a linguistic component.

There are other ways of subdividing the contents of consciousness: between sensations and conscious thoughts (Block, 1995; Bayne and Montague, 2011), between phenomenal (P) and access (A) consciousness (Block, 1995), or core (CC) vs. extended (EE) phenomenal states in consciousness state space (Berkovich-Obana and Glicksohn, 2014), or through choosing a conservative vs. a liberal stance (Kemmerer, 2015). Most of these capture the distinction I’ve made above in one form or another, but for my purposes it is less important to determine where precisely the dividing line is drawn than to ensure that formats are excluded from consideration for being inherently too complex to map in a simple fashion. This avoids some of the conceptual difficulties highlighted by Velmans (2009), including the distinction between qualia and the reflexive or self-referential awareness of those qualia (Peters, 2014), and the “level” of consciousness is likewise not relevant (Overgaard and Overgaard, 2010; Bayne et al., 2016), as it might be affected by, say, sleep or anesthesia, so long as the qualia in question are unaltered in their character.

Treating qualia as more fundamental than more complex contents does not mean qualia necessarily evolved first. In fact the opposite would be the case if, as in many theories, the emergence of consciousness in evolution depended on algorithmic processes, e.g., of sensory processing, episodic memory or learning. The first content of consciousness would then have had at least some of the properties of format, but the sequence in which contents were added to evolving consciousness is not crucial to this analysis, nor is it a problem if there is some degree of dependence on algorithmic processes for most, if not all, conscious experience. Here I require only (1) that the set of all qualia, conceived of as fundamental units of experience, is not the null set, so that it is possible to have qualia that are not inextricably embedded in formats, and (2) that experiences that appear to be simple are indeed so, or at least can be dealt with as such, as qualia rather than formats. Three examples have then been selected that in my view provisionally pass muster in this respect: the simplest of tactile sensations, e.g., a sharp pain or itch (disregarding the means by which these are localized), the frequency range of sound, and the spectrum of light as we perceive it. These are used in the discussion of experience space in the section “Trajectories in Experience Space.” To begin, however, it is necessary to consider how NCCs might be represented in a space that would map to experience space, where again, anything overtly format-like is excluded.

## Selector Circuit Space: Robustness and Routes to Innovation

There are multiple ways of constructing topological spaces to represent the physical factors that contribute to conscious experience: a space for mapping the genomic contribution to neural structure and activity, for example, or an NCC space mapping the neural correlates that underpin conscious experience, either as structural variables, activity-based variables or both. Because the genomic determinants of neural structure and activity are so far removed from the immediate mechanisms that evoke consciousness, the focus here is at the level of NCCs. The analysis could equally well be applied to any neural function, not just consciousness, excepting that, whereas there are various ways to model non-conscious neural circuits based on known examples, the absence of any consensus regarding what NCCs actually look like means that for consciousness, an indirect approach is currently the only available option.

A generally accepted definition of NCCs by Chalmers (2000) employs the idea of a mapping between the physical and the experiential: that NCCs are a “minimal neural system N such that there is a mapping of N to states of consciousness…” with caveats being that we need to be cognizant of whether N is both necessary and sufficient, or only the latter (Fink, 2016), and that correlates are not confused with markers or constituents of consciousness (Michel and Lau, 2020). Here I restrict the analysis to a subset of NCCs that I will refer to as selector circuits (SCs), defined as the neural circuits or activity patterns that serve as the proximate cause that a particular experience is evoked rather than some other. SCs would then fit into previously defined categories of core correlates (Block, 2005), differentiating NCCs (Hohwy and Bayne, 2015), and difference makers of consciousness (DMCs, see Klein et al., 2020).^2^ Klein et al. (2020) make the case for choosing difference makers over NCCs in the broad sense for their greater utility for dealing with causation in complex multi-component systems, the emphasis being on those correlates responsible for changing system output in a predictable way. Change is equally central to the conception developed by Neisser (2012) for isolating the causal component from an otherwise causally neutral set of neural correlates, a task that will be increasingly important as real data begin to emerge on NCC circuitry in real brains.

The kind of topological mapping I propose for SCs formally resembles one used by Fink (his figure 3, which maps “neural events”), but is more precisely defined, as a map of all possible configurations of those categories of circuits capable of acting as SCs, including variants that do not evoke an experience as well as those that do. The latter will then appear as islands, or domains, one for each experience, surrounded by a sea of variants with no selective effect on experience (Figure 1). SC-space is treated as a metric space, in which distance has an explicit meaning: distance between two adjacent points on the map will be defined as the smallest incremental change in the way SCs can be configured, or in the case of circuit activity, the smallest incremental change in the dynamical properties of the SCs in question. The cause of such changes could be genomic, e.g., due to mutation and recombination, or arise from variations introduced during brain development. I require here only that the incremental changes are observables of the system, available to a privileged observer to whom all physical features of the system are known, and are quantifiable, at least in principle. Thus, proximity in SC-space equates to similarity in neural structure or activity patterns, and proportionately greater distance reflects incrementally greater differences in these same variables. Expressing this in a two-dimensional map is clearly inadequate when even a moderately complex neural circuit will have myriad structural and activity-based features that can be configured in many different ways. The system then has many degrees of freedom that can only be fully captured in an n-dimensional space for very large n. Here, for purposes of illustration, *n* = 2 will suffice, with the caveat that there will be artifacts of this compression, e.g., that much of the incremental character of changes in higher dimensional space may be lost when mapped to one of lower dimension.

Consider next, with reference to Figure 1, how an SC for a given experience would be represented: as a single point in SC-space or a grouping of points. There would be a single point if, for an individual brain, the experience in question was evoked by either a single neural event or a set of exact, simultaneous replicates of that event. But if multiple events are required that exhibit some degree of variation, e.g., in the precise architecture of the circuits involved, the timing of events, or any other feature that makes them less than identical and simultaneous, the result is a point cloud. The position of the point (for a single event) or the point cloud (for multiple distinguishable events), and the degree of dispersion of the point cloud will, in the real world, vary between brains. In consequence, the experience evoked can potentially vary as well, so that a pinprick, for example, would be experienced differently from one individual to another, but each would still recognize the experience as painful. The key issue then, from an evolutionary perspective, is to determine which distribution of points in SC-space is most robustly buffered against being degraded over evolutionary time, that is, from generation to generation. The same question applies at the population level, where the SCs would necessarily map as a point cloud representing variation across the population. The consequences of occupying a less-than-optimal location in SC-space are different in these two cases, however. For an individual brain, a shift in position in SC-space will directly affect the experience, e.g., by enhancing, degrading or abolishing it. At the population level, this translates into an increased incidence of either enhanced or impaired experience across the population as a whole, and increased or reduced fitness for some individuals as compared with others.

Consider the case of an individual brain in more detail. We do not know how much mechanistic redundancy is built into the circuitry involved in sensory processing and consciousness (Hohwy and Bayne, 2015), but assuming there is some, the result in SC-space is a point cloud that, if highly localized, produces a combined experience that sums the separate contributions of component circuits that are nearly identical. For a more dispersed cloud there is a greater chance that the resultant experience combines components that are significantly different in character (e.g., that experience **A** in Figure 1 might differ significantly from **A^∗^**). Having a larger and more disperse point cloud thus risks degrading the experience for an individual brain because some SCs will be altered to the point where they either make no contribution to the experience or introduce an element belonging to some distant variant of that experience. Assuming this is disadvantageous, selection will act to minimize the likelihood of it happening, giving localized point clouds an evolutionary advantage over larger diffuse ones. In consequence, the SCs produced over evolutionary time by real brains should map to a progressively shrinking subregion within their respective domains, at both the individual and population level, as they are extinguished from regions near domain boundaries (shown in pale colors in Figure 2). Redundancy is also a consideration. If there is little redundancy, meaning one or a few SC variants are required per brain to evoke an experience, then the reliability of the result depends on those few SCs being precisely replicated in each generation. With greater redundancy, meaning larger numbers of SCs, the deleterious effect of a few of these either degrading or otherwise altering the experience is reduced. Hence redundancy, coupled with stabilizing selection, will buffer the system against the maladaptive randomizing effects of mutation, recombination, and developmental variation as these impinge on individual SCs. Data on real SCs should also then show a positive correlation between the fraction of the potential domain to which those SCs map and the tightness of control exercised over their development.

Figure 2 illustrates the above arguments graphically using three domains (A, B, and C) representing regions in SC-space where SCs localized to A and B evoke, respectively, distinguishable experiences **A** and **B**. For a large domain, many different SC variants would map to the same experience. Whether large domain size is advantageous in and of itself, natural selection has no way of controlling this because domain size for a particular experience is an ontological given, belonging to the realm that Godfrey-Smith (2019), for example, refers to as “the physical.” But what evolution can do is adjust the fraction of the domain that is occupied by the SCs of real brains. Whether the SCs act singly or in combination, what this means in practice is that SCs too near domain boundaries will be progressively eliminated, because small changes in map position alter the experience evoked (arrows from X in the left panel, which either abolish **A** or convert it to **B**) more easily than more distant points (arrow from Y), making the former less robust to genomic change and developmental variation. Assuming evolution favors robustness, the SC variants that survive selection will occupy a progressively smaller proportion of the original domain, so the point clouds of SCs formed by real brains both diverge and are reduced in size as shown in right panel.

But how would such domains arise in close proximity in the first place? Since only small changes in configuration are needed to alter the experience evoked, the underlying mechanism for evoking **A** and **B** would in such cases be similar, sharing many common features. The implication is that A and B are evolutionarily related, raising the possibility that they arose by common descent from an ancestral domain whose SCs once evoked an undifferentiated combination of **A** and **B** together. Refining this ancestral experience (an ur-quale in this formulation) so that **A** and **B** diverge, would have meant selecting brains where the activity of SCs mapping to A are increasingly correlated with each other, but not with those localized to B, and vice versa, and arranging for behavior to depend on this difference. By way of example, suppose one of the degrees of freedom represented by distance across SC-space relates to the timing of relevant neural events, e.g., either in frequency or duration. What we would then see is one set of frequencies or durations evoking **A** more than **B**, and eventually, by selection of variants, evoking **A** to the exclusion of **B**. By this means an initially large SC domain could, in principle, be repeatedly subdivided to produce a range of progressively more refined and precisely specified experiences.

Domain C is included in Figure 2 as a reminder that there is a second route toward innovation, by addition and combinatorial action rather than subdivision. If we think of SC-space as defined so as to represent all possible SCs, evolution is, in effect, exploring a configuration space where any point in that space potentially represents a novel circuitry variant that would either alter an existing experience or evoke an entirely new one. Thus, C could evoke novel experience **C**, or A and C acting together might evoke that same **C**. Further, there could be any number of such distinct C-like domains, i.e., D, E, F, and so on, acting in combinatorial ways, and they need not be linked by descent. Encountering them allows evolution to expand the range of qualia that are experienced, while ensuring at the same time that they are robustly isolated from one another in terms of distance across SC-space. This is especially the case for new domains in distant parts of an n-dimensional SC-space, because the circuitry involved would then be well separated from other SCs by many configurational differences.

Of the various ways qualia might diverge from one another over evolutionary time, Figure 3 shows two ends of a spectrum of possibilities, and can be interpreted as applying either at an individual or population level. However, at the individual level it is more meaningful (and this account will assume) that we are dealing with a situation of high redundancy, i.e., where multiple SC replicates act in concert. We can then have a situation, as in Figure 3A, where the ur-quale is evoked by a point cloud of SCs distributed over a large domain capable of evoking a multiplicity of qualitatively different sensations combined together in a single resultant experience. The sequence of progressive refinement would follow what I have chosen to call the evaporating puddle scenario (“puddle” for short), by analogy to the uneven evaporation of a large shallow puddle, leaving smaller residual puddles behind within the original outline. By analogy, in this scenario, as evolution progressively eliminates some SCs, those that remain would respond to sensory input by evoking a progressively more restricted set of qualia, each representing an element of experience present in the ur-quale from which they all derive. An example might be an ur-quale that, in this ancestral condition, combined together an assortment of negative feelings, such as fear, anxiety, panic, despair and disgust (see Panksepp, 2016) that come to be experienced separately by more highly evolved brains. The second alternative is the tree scenario (Figure 3B) where the SC variants are more tightly clustered from the outset, in a small domain, so as to produce an ancestral ur-quale of a more restricted kind. Over time, the original domain could then spawn sub-domains that diverge, like branches from a stem, so that the new experiences evoked by the SCs in each subdomain become realized contents. The experiences themselves are then well defined throughout, but change incrementally in character as evolution explores surrounding regions of SC space. Because the SC point cloud is small from the start, a higher degree of developmental precision would be required throughout this branching process compared with the puddle scenario. Also, since the tree fans outward over time, novel, divergent experiences can evolve that differ in significant ways from the ur-quale.

One can then ask, of all the qualia we experience, how many, if any, trace their origins to patterns of the above kind, and hence are related through homology. A plausible conjecture is that this is most likely to be the case for qualia sharing related sensory modalities. Obvious examples would be sets of related emotional states, e.g., the negative feelings referred to above, the different acoustic tones we hear, or the spectral colors that arise in vision. One can also ask, since SC-space is a configuration space rather than a real space, if this analysis provides any clues about the number of neurons or volume of tissue required to implement a set of SCs. The answer is that it does not, because the physical volume occupied by the configuration representing a given point in SC-space, whether large or small, is not specified. Consequently this account makes no claims about the actual size, structure or complexity of SCs, and includes no circuitry diagrams, because there is no way currently to choose between many possible options. SCs could be subcomponents of large diffuse cortical networks, or small localized circuits of a few neurons; they could depend on structural features such as the way active synapses are deployed in 3D space, or on activity patterns where it is the pattern itself that exerts a selective action. What can be said is that redundancy matters, and if multiple SCs of similar type must act in concert, implementing this should require a greater volume of tissue than if there is no such redundancy.

Finally, recall that for real populations, there is the problem of maintaining an optimal set of SCs from generation to generation against the degrading effects of random genomic and developmental events. It is a matter of conjecture how rapidly, in the absence of selection, this would happen, but there is no reason that the rate should be the same for both simple and complex contents, i.e., for qualia as compared to the more complex experiences I have here categorized as formats. For qualia, the issue is how reliably some SC variants are formed rather than others. In contrast, for formats, robustness depends on the reliability of reproducing, in each generation, the circuits that execute the algorithms on which each format depends, which are almost certainly different from, and independent of the SCs responsible for evoking the qualia themselves. Hence there is a real possibility that formats can be more robust than the qualia they employ. This could have practical consequences where formats have come to dominate behavioral decision-making, as they have for our own species. In this sense, the distinction made here between qualia and formats is important, not only as a theoretical construct, but for its possible real-world implications.

## Trajectories in Experience Space

The SC-space considered above is a way to represent the measurable properties of a real physical system, i.e., of circuits and their activity. E-space is different in attempting to represent the non-physical properties of experience itself. It is not then a configuration space in the usual sense, as there is nothing physical to configure, but it is a metric space where distance has a specific meaning: that each increment in distance is the minimal distinguishable difference between two experiences. To avoid complicating this definition with issues of a strictly subjective nature, such as whether one can be sure that two different tones of sound are as distinguishable from each other as either is from a flash of light, I will add the further criterion that any two adjacent points are those between which no experience can be inserted that is not intermediate between the two. So, for example, the only experience between two acoustic tones would be another acoustic tone, which disallows a flash of light or a noxious odor from occupying that location. In practical terms, this means adjacent points will belong to sensory experiences that are either the same or only incrementally different. This raises the point of whether different qualia in fact share features that allow them to be mapped together on the same surface, a question which, as discussed below, may depend on whether they are related through homology.

Other topological constructs have been used to investigate conscious experience. Of these, E-space as defined here differs from quality space, which maps subjective experience quantitatively, and which from my perspective is problematic when applied to complex sensory states, such as vision (see critique by Matthen, 2004), but also in other applications (Kostic, 2012; Young et al., 2014). I likewise distinguish between E-space and similarity space, which maps experiences with respect to their similarities and differences, as my concern is less with the relation between physical stimuli and the sensations they evoke than with how, in principle, neural circuitry acts to shape subjective experience.

To this end, E-space is treated here as the space of all possible qualia regardless of whether they are experienced by any particular brain. This is meant simply as a convenience for this particular thought experiment, not to argue in support of the view that the ultimate source of conscious experience lies outside the biological realm. It also means that E-space will be larger than the subdomain available to a given brain, so that evolution can be thought of as acquiring novel qualia as it explores E-space through neural innovation. The human brain, for example, might have the potential for an experience equivalent to a bat’s, during echolocation, by evoking it from regions of E-space that are available to human brains, but have been rendered silent by evolution. Or, it may be that human brains have never had access to those regions of E-space. There could also be many experiences that no vertebrate brain has yet evolved to evoke, but what these might be is, from a human perspective, impossible to judge.

The properties of E-space defined in this way can be illustrated with three examples that map as a point, a line and a closed curve (Figure 4). The first is how I would represent pinprick, which so far as I can see (or, literally, feel), is an experience so simple that, stripped of positional reference, it lacks any other aspect; it simply “is.” For the second, a line, I have chosen the range of tonal sound as registered by the cochlea, where the auditory experience varies in a graded way depending on vibrational frequency, but terminates at some point at both ends. For my purposes it does not matter whether each tone is treated as a distinct quale, or whether sound at different frequencies is a single quale that is “tunable” in some way. What matters is that all other qualia are excluded from the line of tonal experience because they are not intermediate between any two tones. My third example is the visual perception of color, where there is a continuous gradation in the nature of the experience, but no point of termination because the colors, at least as we experience them, form a continuous and recursive sequence (Matthen, 2005, 2020). Combining colors moves you toward the center of the curve, where the color is replaced by white light, so trajectories across the domain enclosed by the curve are graded as required.

The figure shows all three qualia together on the same two-dimensional plane as a way of illustrating a feature that is necessary regardless of how many dimensions the map is intended to represent, and that is divergence. That is, if all three are mapped together, and for the way the metric is defined, the three will be separated by a zone of exclusion surrounding each one (inside the dashed lines in the figure) because qualia too similar to one another risk being indistinguishable in practice, especially at low intensity, which makes them maladaptive from an evolutionary standpoint. It is, after all, at the margins of perception that selection will often exert its strongest effect, e.g., that the antelope that is only slightly less able than other herd members to distinguish between different sensory cues is the one that gets eaten.

The question then is, under what conditions is it appropriate to map diverse qualia to the same topological space. This is ultimately a question about the nature of qualia themselves: are they comparable in kind in the sense that they could in principle grade into one another, or not? With clearly related qualia, such as a set of acoustic tones, one can suppose this is the case, i.e., that they are both similar in character and grade into one another in an a continuum. And it is plausible that they may share a common origin, as an acoustic ur-quale, represented by Q1 in Figure 4. Indeed all experiences of mechanosensory origin (touch, pressure, vibration, hearing) could conceivably derive from a common ur-quale, positioned like Q2 in Figure 4. From this point, the incremental divergence required to evolve the experiences of pinprick and hearing would define a surface by tracing out a trajectory of points in E-space that do in fact exist, because they have existed in the past in real brains. That part of the surface is hence a valid construct in reality. In contrast, considering the qualitative difference between the experiences of light and sound, with no obvious intermediate between them, there is no reason to suppose they could be mapped together. This is reinforced by what we know of the sensory cells involved, that they have evolved from separate receptor-based systems (Schlosser, 2018), so homology between light and sound as experiences is possible, but not expected. Assessing homology can be problematic, however (Hall and Kerney, 2012), the complication here being that judging whether two experiences are homologous based on common descent is quite separate from the issue of homology as it relates to the underlying neural circuits, and these circuits will almost certainly share many common features irrespective of whether the qualia they evoke are homologous at the experiential level.

The advantage of dealing with qualia that are potentially homologous is that a more plausible case can be made for an isomorphic mapping between SC-space and E-space. That is, where patterns of past divergence follow a tree or puddle pattern, E-space might exhibit a matching pattern of diversification and divergence. For sound, for example, the range of frequencies experienced might, in SC-space, be evoked by a continuous sequence of SC domains that map in an orderly fashion to a corresponding line in E-space. This would have the advantage of being a parsimonious explanation, the problem being that we do not know if anything concerning consciousness is, in fact, parsimonious. Evoking new sound experiences across a frequency range might instead depend on the addition of multiple new domains scattered all over SC-space acting in combinatorial ways. Further, distances need not map proportionately, since a short displacement in SC-space could yield a large one in E-space, while a large displacement in SC-space might make no difference at all to the experience. The conclusion is that for qualia sharing common descent, it is possible that there could be an isomorphic mapping between SC- and E-space, but this is by no means the only option.

To conclude this section, it is useful to make a remark on referral, sometimes included among the hard problems of consciousness, e.g., by Feinberg (2012). Take vision, for example, considered here as a format, where the inherent viewpoint ensures that the experience is perceived as external, i.e., it is referred to the outside world (Merker, 2013). The provisional conclusion one might then draw is that referral is a property of any format structured so as to ensure this result, and that other mappings, including the somatosensory map, would share this property. But this is not the only possibility. Consider instead a somatosensory experience that was more akin to the acoustic experience of different frequencies. The conscious sensation of touch at different points along the rostro-caudal axis of the body would then be distinguishable in the same way as acoustic tones generated by the stimulation of different hair cells along the axis of the cochlea. The position-specific aspect of the somatosensory experience would thus be due to a graded or tunable quale, but to a single quale none the less, rather than a format. I mention this as a possibility, not so much to argue the case, but to illustrate the fact that we cannot predict in advance, or even judge from our own experience, the limits of what evolution is capable of doing with the qualia at its disposal.

## Conclusions, and the Function of Consciousness

This account proposes a conceptual framework, using a configuration space analysis, for investigating how evolution acts on the selector circuits (SCs, a subset of NCCs) responsible for evoking a particular conscious content as opposed to any other. The analysis depends on the supposition that there are fundamental units of experiences (qualia) that are distinguishable from more complex contents of consciousness (here, formats), and that qualia can be dealt with individually both at an analytical level and as objects of selection. But there are two further considerations. First, a caveat, that there is good reason to doubt that all contents will yield to the same set of analytical methods, and in particular, that a configuration space applicable to qualia can be usefully applied to formats. And second, a result of the analysis, that the question of evolutionary descent is a significant one, in that qualia that are homologous as experiences are intrinsically more easily dealt with in relation to one another than those that are not. This has practical implications for a future where we have more access to real data on NCCs relevant to various forms of experience, the expectation being that SC-type NCCs will exhibit both constant and variable features, but the variability will be least between qualia sharing common descent.

There is a developmental aspect here as well, since it is the variability among developing brain circuits, and the synaptic plasticity on which this variability depends, that provide the raw material for evolutionary innovation. For consciousness, and for SCs in particular, there are mechanisms that would allow this variability to be harnessed so as to ensure a precisely controlled outcome (Lacalli, 2020). Variability in this case means that the synaptic networks in question can be dynamically reconfigured as they develop, which means a degree of synaptic plasticity is an inherent part of the process. Synaptic plasticity is most frequently dealt with in relation to its role in real-time cortical functions like learning and memory (e.g., Attardo et al., 2015), but for SCs, in contrast, plasticity must diminish at some point during development if the resulting structure is to be stable in real time, and hence produce conscious experiences that are themselves stable. There should consequently be a division of labor among neural circuits, such that those involved in functions requiring real-time plasticity on a continuing basis, like memory, are precluded from involvement in those aspects of consciousness requiring real-time stability, including the evocation of qualia. This has implications for how the different functions associated with the production of conscious sensations are distributed across the brain and its various substructures.

As a final point, the configuration space representation can be used to illustrate something quite precise about the function of consciousness from an evolutionary perspective. I have expressed this previously as follows (Lacalli, 2020, p. 6): that consciousness functions as “a mechanism for restructuring synaptic networks in ways that would not otherwise have occurred, in order to produce advantageous behavioral outcomes that would not otherwise have happened.” Topologically, this is saying that there are regions of SC-space, and hence E-space, that cannot in practice (i.e., in real brains) be accessed except through the agency of natural selection acting on the outcome of consciously controlled behaviors. A consideration of SC-space shows why: that for every region in SC-space that evokes a particular conscious experience, there is a boundary a finite distance away that separates points within that domain from those outside it (cf. Figure 1). Starting from outside the domain, it is possible in principle for a fortuitous change to the genome to move the system in one jump from that starting point to deep within the domain. Hence a specific and reliably evoked quale could theoretically emerge from the non-conscious condition at one jump. But the spatial metric used here means that moving “to deep within” the domain would require multiple changes in the genome, or one change with multiple consequences for development of a very precise type, which means that the chance of this happening randomly is vanishingly small. Evolution achieves this instead through natural selection acting at a population level over multiple generations because, and only because, consciousness has an adaptive advantage over the absence of consciousness at each generational step. Hence, the function of consciousness from an evolutionary perspective is to provide access to otherwise inaccessible points in SC-space (indeed, in NCC-space more generally) and, correspondingly, in E-space. This may seem an unsatisfying conclusion, because it tells us nothing about the proximate purpose for which consciousness evolved, but it is the more general answer, and hence conceptually the more meaningful one.

## Data Availability Statement

There is no data beyond that included in the article; further inquiries can be directed to the corresponding author.

## Author Contributions

TL was solely responsible for the preparation and content of this article.

## Conflict of Interest

The author declares that the research was conducted in the absence of any commercial or financial relationships that could be construed as a potential conflict of interest.

## Publisher’s Note

All claims expressed in this article are solely those of the authors and do not necessarily represent those of their affiliated organizations, or those of the publisher, the editors and the reviewers. Any product that may be evaluated in this article, or claim that may be made by its manufacturer, is not guaranteed or endorsed by the publisher.

## Footnotes

1
https://en.wikipedia.org/wiki/Qualia

2 My choice of SCs over a more neutral term, such as selectors (Ss) or DMCs, in part reflects a mechanistic bias, but also makes the resulting configuration space easier to comprehend and explain.
